# Molecular Epidemiology of Extensively Drug-Resistant *mcr* Encoded Colistin-Resistant Bacterial Strains Co-Expressing Multifarious β-Lactamases

**DOI:** 10.3390/antibiotics10040467

**Published:** 2021-04-20

**Authors:** Hasan Ejaz, Sonia Younas, Muhammad Usman Qamar, Kashaf Junaid, Abualgasim Elgaili Abdalla, Khalid Omer Abdalla Abosalif, Ayman Ali Mohammed Alameen, Mohammed Yagoub Mohammed Elamir, Naveed Ahmad, Sanaa Samir Mohamed Hamam, Eman Hosney Mohammed Salem, Syed Nasir Abbas Bukhari

**Affiliations:** 1 Department of Clinical Laboratory Sciences, College of Applied Medical Sciences, Jouf University, Al Jouf 72388, Saudi Arabia; kjunaid@ju.edu.sa (K.J.); aealseddig@ju.edu.sa (A.E.A.); koabosalif@ju.edu.sa (K.O.A.A.); aaalameen@ju.edu.sa (A.A.M.A.); myelamir@ju.edu.sa (M.Y.M.E.); 2 Department of Pathology, Tehsil Headquarter Hospital Kamoke, District Gujranwala, Kamoke 50661, Pakistan; soniamicro02@gmail.com; 3 Department of Microbiology, Faculty of Life Sciences Government College University, Faisalabad 38000, Pakistan; musmanqamar@gcuf.edu.pk; 4 Department of Pharmaceutics, College of Pharmacy, Jouf University, Sakaka, Al Jouf 72388, Saudi Arabia; nakahmad@ju.edu.sa; 5 Department of Medical Microbiology and Immunology, Faculty of Medicine, Menoufia University, Shebin Elkoum 32511, Egypt; Sanaa.hamam@med.menofia.edu.eg (S.S.M.H.); eman.hosney.salem@med.menofia.edu.eg (E.H.M.S.); 6 Department of Pharmaceutical Chemistry, College of Pharmacy, Jouf University, Sakaka, Al Jouf 72388, Saudi Arabia; sbukhari@ju.edu.sa

**Keywords:** colistin resistance, β-lactamases, extensive drug resistance, plasmid-mediated resistance, *mcr* genes, integrons

## Abstract

Plasmid-mediated colistin resistance (Col-R) conferred by *mcr* genes endangers the last therapeutic option for multifarious β-lactamase-producing bacteria. The current study aimed to explore the *mcr* gene molecular epidemiology in extensively drug-resistant (XDR) bacteria. Col-R gram-negative bacterial strains were screened using a minimum inhibitory concentration (MIC) breakpoint ≥4 µg/mL. Resistant isolates were examined for *mcr* variants, extended-spectrum β-lactamase, AmpC, and carbapenemase genes using polymerase chain reaction (PCR). The MIC breakpoints for *mcr*-positive strains were determined using broth microdilution and E-test strips. Overall, 19/718 (2.6%) gram-negative rods (GNRs) harboring *mcr* were identified, particularly in pus (*p* = 0.01) and tracheal secretions (*p* = 0.03). Molecular epidemiology data confirmed 18/19 (95%) *mcr*-1 and 1/19 (5%) *mcr*-2 genes. Integron detection revealed 15/17 (88%) *Int-1* and 2/17 (12%) *Int-2*. Common co-expressing drug-resistant β-lactamase genes included 8/16 (50%) *bla*_CTM-1_, 3/16 (19%) *bla*_CTM-15_, 3/3 (100%) *bla*_CMY-2_, 2/8 (25%) *bla*_NDM-1_, and 2/8 (25%) *bla*_NDM-5_. The MIC_50_ and MIC_90_ values (µg/mL) were as follows: *Escherichia coli*, 12 and 24; *Klebsiella pneumoniae*, 12 and 32; *Acinetobacter baumannii*, 8 and 12; and *Pseudomonas aeruginosa*, 32 and 64, respectively. Treatment of XDR strains has become challenging owing to the co-expression of *mcr*-1, *mcr*-2, multifarious β-lactamase genes, and integrons.

## 1. Introduction

Multidrug-resistant (MDR) and extensively drug-resistant (XDR) gram-negative bacteria represent significant global public health threats [1]. The contemporary emergence of carbapenem-resistant enterobacteria has dramatically increased the cationic peptide colistin’s reliance, which is commonly considered the last-resort antibiotic [2]. The reinstatement of the older and less user-friendly antibiotic (nephrotoxic and neurotoxic) colistin has occurred as a response to the limited alternative treatment options available against MDR bacteria [3]. However, as an ultimate antimicrobial drug, colistin is impaired by the emergence of mobile colistin resistance (*mcr*) genes [1].

In 2016, *mcr-1* was initially reported among *Enterobacteriaceae* isolated from humans and food-producing animals in approximately thirty territories over five continents [1,4]. The unbridled use of colistin in agriculture and the increased demand for colistin in clinical practice resulted in the rapid propagation of resistance [2]. Acquired colistin resistance (Col-R) has been observed in *Klebsiella pneumoniae*, *Acinetobacter baumannii*, *Pseudomonas aeruginosa,* and some other bacterial genera. Inherent resistance to this class of antibiotics has been identified in some strains of *Proteus*, *Serratia*, *Neisseria*, *Burkholderia,* and *Providencia* [5].

Col-R relies on a reduction in the electrostatic attraction between colistin and the outer membrane of gram-negative bacteria [2]. The *mcr* genes diminish bacterial affinity toward colistin by encoding phosphorylethanolamine transferase, which reduces the negative charge of the microbial outer membrane, resulting in the development of microbial resistance [6]. The increased detection of *mcr* in bacterial strains has shifted the paradigm of MDR to XDR for several bacterial strains, and the dissemination of *mcr* appears to be associated with the rapid horizontal transmission of plasmids [7]. Several *mcr* genes (*mcr-1* to *mcr-9*) have been described during the last four years from different countries [6].

The occurrence of the most commonly isolated *mcr* gene, *mcr-1,* has been reported in species of *Escherichia coli* and *K. pneumoniae* isolated from poultry, meat, and humans [1]. Poultry is the most frequent source of *mcr-1*-harboring bacteria, which has been isolated from various phases of poultry development [8]. Resistance mediated by *mcr-1* has also been reported in bacteria isolated from humans, poultry, retail meat, pigs, pigeons, ducks, and geese in China [1]. Extended-spectrum β-lactamase (ESBL)-producing bacteria represent a major health concern worldwide. The most common ESBL variants include *bla*_TEM_*, bla*_SHV_*,* and *bla*_CTX-M_, which are considered major causes of hospital and community infections [9]. AmpC β-lactamase (AmpC) is another class of β-lactamase, which differs from ESBLs owing to their ability to hydrolyze cephamycins, and AmpCs are not affected by available β-lactamase inhibitors [10]. The most abundant plasmid AmpC found in *E. coli* is CMY-2, which has been recorded in various geographical areas, including Asia, North America, and Europe. In particular, the occurrence of Col-R in ESBL or carbapenemase-producing bacteria poses a serious health risk owing to the limited therapeutic options available for the treatment of these strains [11]. The plasmids containing *mcr-1* have been identified in MDR *Enterobacteriaceae* isolates, including species that produce carbapenemases, such as *K. pneumoniae* carbapenemase (KPC), Verona integron-encoded metallo-β-lactamase (MBL)-producing (VIM) species, or New Delhi MBL (NDM)-producing species [12]. Since the discovery of NDM-1 in India, over 24 NDM variants have been identified [13]. The co-existence of *mcr* and NDM has been reported in specimens isolated from food-producing animals and clinical samples, increasing the public health burden associated with antimicrobial resistance [14].

The emergence of plasmid-mediated *mcr* bacterial species in animals and humans is an overwhelming and current problem that has jeopardized public health and may lead to the development of virtually untreatable infections. The present study aimed to explore the molecular epidemiology of *mcr* genes and rule out the co-existence of ESBLs, AmpCs, and carbapenemases in *mcr* encoded bacterial strains. This study’s findings will help in understanding the co-existence of drug-resistant genes, the menace posed by horizontal gene transfer, and the minimum inhibitory concentrations (MICs) required for antibacterial drugs to treat these bacterial strains.

## 2. Results

### 2.1. Demographic Characteristics of Patients Infected with Col-R Strains

**Figure 1:**
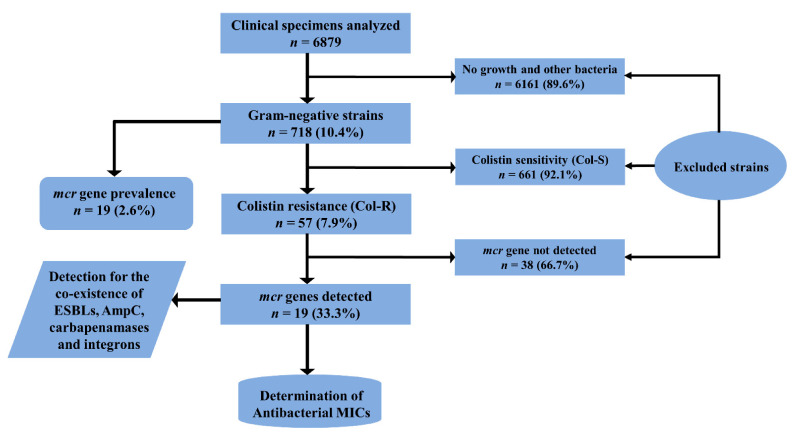
Flow diagram showing the collection of clinical specimens, the isolation of bacterial strains, and the inclusion and exclusion of bacterial strains in the study. ESBL, extended-spectrum β-lactamase; MIC, minimum inhibitory concentration.

A total of 718 gram-negative, non-duplicate strains were evaluated for colistin resistance, which resulted in the identification of 57 (7.9%) Col-R and 661 (92.1%) Col-S strains. Overall, *mcr* genes were detected in 19 (2.6%) gram-negative strains, which represented 33.3% of the total Col-R strains (Figure 1). The frequency of *mcr*-encoded strains was higher in women than in men; however, the statistical analysis indicated no significant association (*p* = 0.78) between the sex of the patients and the collection of *mcr*-positive isolates. The highest number of *mcr* genes was detected from the isolates obtained from the medical ward and intensive care unit (ICU), followed by nephrology, the outpatient department (OPD), and the orthopedic ward. However, only specimens obtained from the ICU and surgical wards (*p* = 0.01) were significantly associated with the presence of *mcr* genes. Among all sources, *mcr*-harboring isolates were significantly associated with pus (*p* = 0.01) and tracheal secretion (*p* = 0.03) specimens (Table 1).

### 2.2. Distribution of Col-R and Col-S Bacterial Strains

**Figure 2:**
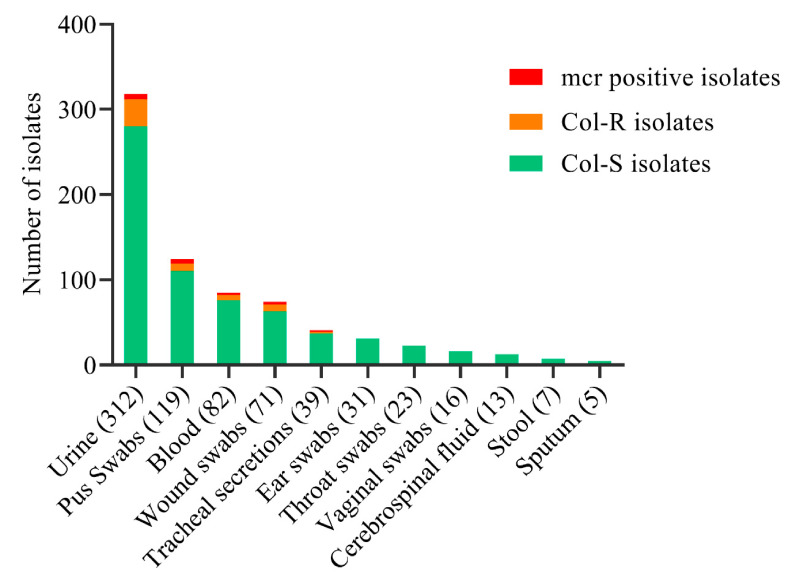
Distribution of colistin-sensitive (Col-S), colistin-resistant (Col-R), and *mcr* genes among the gram-negative bacterial strains isolated from various sources (*n* = 718).

Overall, 17/341 (5%) *E. coli*, 9/185 (4.9%) *K. pneumoniae*, 4/52 (7.7%) *A. baumannii*, 2/90 (2.2%) *P. aeruginosa*, and 25/25 (100%) *P. mirabilis* were identified as Col-R species. No significant association was observed between Col-R and *mcr* gene-harboring strains. We detected *mcr* genes in 9/17 (52.9%) *E. coli* (*p* = 0.43), 5/9 (55.6%) *K. pneumoniae* (*p* = 0.78), 3/4 (75%) *A. baumannii* (*p* = 0.43), and 2/2 (100%) *P. aeruginosa* (*p* = 0.49) Col-R species. None of the *P. mirabilis* strains were positive for *mcr* genes; however, these strains were found to be Col-R owing to intrinsic resistance (Table 2). Col-R specimens were obtained from 32/312 (10%) urine samples, 9/119 (7.6%) pus swabs, 6/82 (7.3%) blood samples, 8/71 (11.3%) wound swabs, and 2/39 (5.1%) tracheal secretions. Similarly, *mcr* sources were observed in 6/312 (1.9%) urine samples, 5/119 (4.2%) pus swabs, 3/82 (3.7%) blood samples, 3/71 (4.2%) wound swabs, and 2/39 (5.1%) tracheal secretions (Figure 2).

### 2.3. Molecular Detection of mcr and β-Lactam Drug-Resistant Gene Variants

The molecular analysis detected two *mcr* variants among the Col-R strains. Overall, 18/19 (95%) strains harbored *mcr*-1, and 1/19 (5%) strain harbored *mcr*-2 among all Col-R clinical isolates. The source of the only isolate carrying *mcr*-2 was a tracheal secretion isolated from an ICU case. All *mcr*-positive strains co-expressed drug-resistant β-lactamase genes. The most common ESBL-producing gene variants were 8/16 (50%) *bla*_CTM-1_ and 3/16 (19%) *bla*_CTM-15_. The AmpC gene variant *bla*_CMY-2_ was detected in 3/3 (100%) strains. Carbapenemase-producing strains included 2/8 (25%) *bla*_NDM-1_, 2/8 (25%) *bla*_NDM-5_, and 1/8 (13%) each of *bla*_IPM_, *bla*_OXA-48_, *bla*_OXA-51_, and *bla*_VIM_, respectively. Notably, 17 bacterial strains co-harbored integrons, of which 15/17 (88%) were *Int1* and 2/17 (12%) were *Int-2* (Table 3).

### 2.4. Antibacterial Resistance Spectrum in mcr-Positive Bacteria

**Figure 3:**
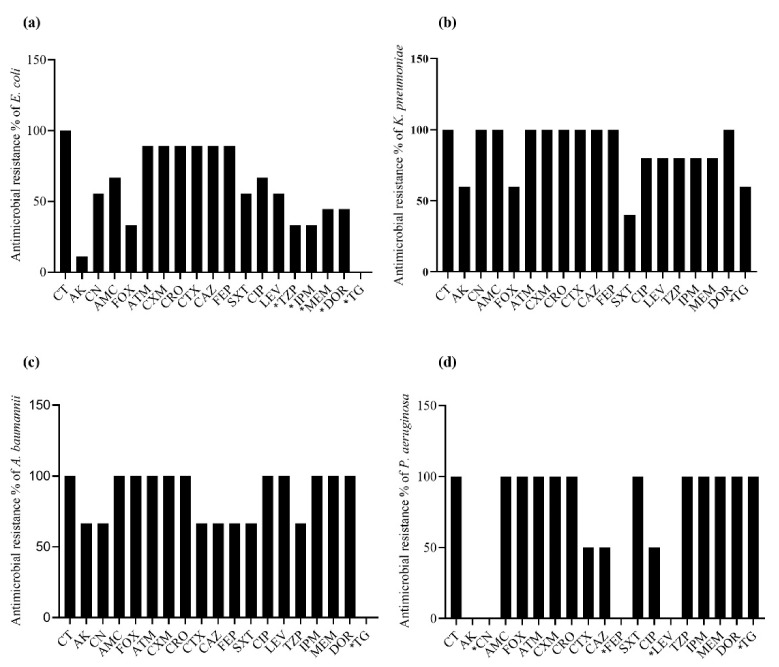
Resistance profile of *mcr*-harboring gram-negative bacterial isolates (*n* = 19). Figure 3a–d show the resistance spectrum of *E. coli*, *K. pneumoniae*, *A. baumannii* and *P. aeruginosa*, respectively. CT, colistin; AK, amikacin; CN, gentamicin; AMC, co-amoxiclav; FOX, cefoxitin; ATM, aztreonam; CXM, cefuroxime; CRP, ceftriaxone; CTX, cefotaxime; CAZ, ceftazidime; FEP, cefepime; SXT, co-trimoxazole; CIP, ciprofloxacin; LEV, levofloxacin; TZP, piperacillin-tazobactam; IPM, imipenem; MEM, meropenem; DOR, doripenem; TG, tigecycline. * Significant *p*-values < 0.05.

Overall, the spectrum of MCRPBS showed extensive drug resistance against several antibiotics. A total of eight (88.9%) *E. coli* strains showed resistance to aztreonam, cefuroxime, ceftriaxone, cefotaxime, ceftazidime, and cefepime. Four (44.4%) *E. coli* isolates were resistant to each of meropenem and doripenem, and three (33.3%) were resistant to piperacillin-tazobactam and imipenem. None of the isolates showed resistance to tigecycline (Figure 3a). All of the *K. pneumoniae* strains were resistant to cephalosporin, aztreonam, co-amoxiclav, gentamicin, and doripenem. A total of three (60%) strains were resistant to amikacin, cefoxitin, and tigecycline, and only two isolates were resistant to (40%) co-trimoxazole (Figure 3b). Extensive drug resistance was also observed in the *A. baumannii* isolates; however, none of the *A. baumannii* isolates were found to be resistant to tigecycline (Figure 3c). All *P. aeruginosa* isolates were resistant to piperacillin-tazobactam, carbapenems, tigecycline, and several cephalosporin drugs. Despite drug resistance to several antibiotic classes, *P. aeruginosa* expressed no resistance to gentamicin, cefepime, and levofloxacin (Figure 3d).

### 2.5. MIC_50_ and MIC_90_ in mcr Gene-Harboring Bacterial Strains

The MIC to inhibit 50% growth (MIC_50_) and the MIC to inhibit 90% growth (MIC_90_) were observed using colistin (breakpoint ≥ 4 µg/mL) and other antibiotic groups, based on their respective breakpoints. The colistin MIC_50_ and MIC_90_ values were as follows: *E. coli*, 12 and 24 µg/mL; *K. pneumoniae* 12 and 32 µg/mL; *A. baumannii* 8 and 12 µg/mL; and *P. aeruginosa* 32 and 64 µg/mL, respectively. The MIC_50_ and MIC_90_ of all the isolates against each tested drug are listed in Table 4.

## 3. Discussion

The recent appearance of plasmid-mediated Col-R *Enterobacteriaceae* has drawn remarkable attention globally because this emergence has resulted in the deterioration of the last-resort antimicrobial commonly used to treat XDR bacterial infections, leading to reports of dramatic colistin inefficacy in several cases. This study examined 718 clinical isolates and identified 57 (7.9%) as Col-R and 661 (92.1%) as Col-S. These findings are consistent with a previous report from Colombia, in which (8.7%) Col-R was detected among clinical isolates [15]. The data from various research studies have reported a range from 0% to 31.7% Col-R [16,17] strains, which could be due to differences in factors such as poor infection control practices, the extensive use of colistin, and differences in the methodologies used to detect resistance. The literature suggests a lower prevalence of *mcr*-positive bacterial strains (MCRPBS) among human sources than among animal sources, which could indicate that plasmid-mediated colistin resistance first evolved in animal strains and then transferred to humans [1].

The prevalence of *mcr* in our study was 2.6%, similar to an earlier report, which showed a prevalence in Pakistan of 2.8% [18]. The findings of studies performed in other countries correspond well with those of our study, with studies in Iran and India reporting *mcr* rates among clinical isolates of 3% and 3.2%, respectively [19,20]. The *mcr-1* gene is most commonly found in *E. coli* compared with other bacterial species globally [21,22]. We identified nine *E. coli*, five *K. pneumoniae*, three *A. baumannii,* and two *P. aeruginosa* isolates harboring *mcr* genes, which are similar proportions as those reported by a study performed in Korea [23]. Our findings show the wide distribution of *mcr*-harboring *E. coli* isolates, primarily recovered from urine, followed by pus swabs, blood, wound swabs, and tracheal secretions, which agrees with previous reports [24,25]. The frequency of *mcr* genes among clinical isolates was higher among women than men, consistent with an Iranian study [25]. There is no significant relationship between *mcr* detection and sex, and the only explanation for the higher occurrence in females is associated with the source of MCRPBS. Most of the *mcr*-harboring isolates were detected from the urinary samples, and urinary tract infections occur more frequently in women.

The molecular analysis showed two *mcr* variants among the Col-R strains, 95% *mcr-1* and 5% *mcr-2*, consistent with global surveillance reports in which *mcr-1* was detected in 75.8% of isolates from 18 countries [20]. Colistin has been used as the last-option antibacterial to treat XDR infections, but its efficacy has reduced since the emergence of *mcr*-positive strains [26], which has greatly compromised the therapeutic strategy for addressing MDR strains. MCRPBS in our study expressed extensive drug resistance to several classes of antibiotics. *E. coli* strains were resistant to aztreonam, cefuroxime, ceftriaxone, cefotaxime, ceftazidime, and cefepime and presented a variable spectrum of resistance to other classes of drugs. *K. pneumoniae* strains emerged as MDR and presented resistance to cephalosporin, aztreonam, co-amoxiclav, gentamicin, and doripenem. The findings of our study are consistent with those of an earlier report [25]. *A. baumannii* showed extensive drug resistance, and the diverse XDR strains had been reported by other studies [27,28,29].

The expanded drug resistance to β-lactams, aminoglycosides, carbapenems, and other antimicrobial drugs poses a significant global threat [30,31]. We found that high proportions of *mcr*-harboring isolates were characterized with an MDR profile, with particular resistance against third-generation cephalosporins. The expression of ESBLs and AmpC can explain the expansion of cephalosporin drug resistance, which is consistent with previously reported findings [20]. MCRPBS co-expressed ESBL gene variants (*bla*_CTM-1_ and *bla*_CTM-15_), one AmpC gene variant (*bla*_CMY-2_), and carbapenemase-producing genes (*bla*_NDM-1_, *bla*_NDM-5_, *bla*_IPM,_ *bla*_OXA-48,_ *bla*_OXA-51_, and *bla*_VIM_). These results agree with a previous report, in which 50% of *mcr*-harboring *E. coli* strains were found to be resistant to third-generation cephalosporins owing to the co-expression of *bla*_CTX-M-2_, and five isolates also expressed *bla*_NDM_ and *bla*_KPC_ [24]. Our study shows the co-existence of *mcr-1* and *bla*_NDM_ in several strains, which agrees with a previous report [32]. This study found one strain of *P. aeruginosa* co-harboring *mcr-1* and *bla*_NDM-5_, which was recovered from urine, similar to a previous study in which a strain of *E. coli* co-expressing *mcr-1* with *bla*_NDM-5_ was recovered from urine [33]. The most common ESBL-producing gene variants were 8/16 (50%) *bla*_CTM-1_ and 3/16 (19%) *bla*_CTM-15_, suggesting the possible dissemination between humans and animals owing to selective pressure between the animal and human environment.

Integrons contain a drug-resistance gene cassette that can act against many drug categories and represent a core component of multidrug resistance. We found 17 bacterial strains that co-harbored integrons, of which 88% were *Int-1* and 12% were *Int-2*. Integrons are active in the development and dissemination of antibiotic resistance in gram-negative pathogens [34]. One limitation of the present study was that we focused on the most commonly reported *mcr*-*1* to *mcr-5* variants. We were unable to examine all of the *mcr* variants and sub-variants in the bacterial strains of clinical significance owing to limited resources.

## 4. Materials and Methods

### 4.1. Study Design and Ethics Approval

Bacterial strains were collected prospectively from various clinical settings located in Faisalabad and Lahore, Pakistan. The study design followed the ethical principles described by the World Medical Association (WMA) and the Declaration of Helsinki [35]. The study was performed as a collaboration between Government College University Faisalabad and Jouf University Saudi Arabia. The institutional review bodies for both institutions issued ethical approval for the analysis of bacterial strains. No human or animal trials were conducted during the study. Informed consent was not necessary because the strains were collected from human samples, but not associated with any individual patient data.

### 4.2. Specimen Collection and Processing

A total of 6879 clinical specimens were collected over six months from various sources and examined for bacterial isolation. The patients’ sources include blood, urine, pus, tracheal secretion, cerebrospinal fluid (CSF), stool, and different swabs. No environmental swabs or water samples from the hospital environment were included for analysis. Blood and MacConkey’s agar were used to culture all clinical specimens, except for blood and urine specimens. The blood samples were inoculated first in brain heart infusion broth. After a period of incubation following bacterial growth indicators, these cultures were subcultured on blood and MacConkey’s agar. The urine specimens were processed on cystine–lactose–electrolyte-deficient (CLED) agar. All the cultures were incubated at 35–37 °C overnight in an aerobic incubator.

### 4.3. Bacterial Growth and Characterization

Bacterial strains were phenotypically (growth characteristics) and biochemically characterized using Gram’s stain, conventional biochemical tests (catalase, oxidase, urease, and indole), and analytical profile index (API) 20E and 20NE (bioMérieux). Gram-negative rods (GNRs) were selected for further identification, and the remaining cultures were excluded from the analysis (Figure 1). Urine cultures showing GNRs with >10^5^ colony-forming units (CFU)/mL were considered significant bacteriuria.

### 4.4. Resistance Profile and MIC Determination

The Col-R status of the retained GNRs was detected using SensiTest™ Colistin (Liofilchem, Via Scozia, Italy). Bacteria exhibiting MICs ≥4 µg/mL were phenotypically reported as Col-R strains. MCRPBSs were tested for MDR and XDR. MICs were determined against several antibacterial drugs using the broth microdilution method and *E*-test strips (Liofilchem, Via Scozia, Italy), and the inoculum size was standardized using a 0.5 McFarland standard. The tested antibacterial drugs included cephalosporin, fluoroquinolones, carbapenems, aminoglycosides, and β-lactam combined with colistin to determine co-resistance. The established MIC breakpoints were used to interpret the results as resistant and susceptible bacterial strains [36].

### 4.5. Screening of ESBLs, AmpC, and Carbapenemases

MCRPBSs were phenotypically characterized to detect the presence of other drug-resistant enzymes (ESBLs, AmpC, and carbapenemases). Phenotypically, ESBLs were identified by the hydrolysis of cefotaxime and ceftazidime and the formation of keyhole effect when using the conventional double-disk synergy technique [37,38]. AmpC was characterized by cefoxitin resistance and the enhancement of the inhibitory zone when using a combination of cefoxitin and boronic acid (inhibitor-based) compared with cefoxitin alone [39]. The isolates were screened for carbapenem resistance and subsequently confirmed for the presence of MBL by the zone enhancement in the presence of ethylenediaminetetraacetic acid (0.5 M EDTA) combined with a carbapenem in the disk-diffusion assay compared with carbapenem alone. Further confirmations were performed using the modified Hodge test [40,41].

### 4.6. Molecular Characterization of mcr Genes

Col-R strains were selected for the analysis of *mcr*-positive bacterial strains (MCRPBSs), and the presence of most frequently isolated *mcr-1* to *mcr-5* variants was detected. We used previously described primers and well-optimized multiplex polymerase chain reaction (PCR) conditions to detect the presence of *mcr* genes [8,42,43]. MCRPBSs were subcultured on nutrient agar to refresh the bacterial growth, and a few colonies from overnight strains were mixed in 500 µL TE buffer following the 0.5 McFarland standard. The suspension was placed in an Eppendorf tube, the caps were sealed with Parafilm to prevent accidental opening, and the tubes were boiled for 15 min in a preheated (100 °C) water bath. The mixture was centrifuged for five minutes at 14,000 rpm/min, and the supernatant was used to perform gene amplification [42]. A 50 µL reaction mixture was prepared for each specimen using 0.5 µM forward and reverse primers (*mcr*-1, 2, 3, 4, and 5), 2 µL template, 25 µL master mix, and 1.5 µL dimethyl sulfoxide (DMSO), and the final reaction mixture was brought to 50 µL using Milli-Q water. Amplified gene products were detected on agarose gel electrophoresis using 6× loading dye at 90 V for 50 min. A 100 bp DNA ladder was used to quantify the gene products, and a gel documentation system was used to visualize the genes.

### 4.7. Molecular Characterization ESBLs, AmpC, Carbapenemases, and Integrons

MCRPBS DNA was extracted as described for *mcr* sequencing and used to amplify drug-resistant genes. ESBL, AmpC, carbapenemases, and integron genes were separately amplified separately using previously described primers and optimized PCR conditions [13,38,44]. The amplicon was preserved at −20 °C for further analysis. DNA sequencing was performed using Big Dye v.3.1 (Life Technologies, Carlsbad, CA, USA) and ABI (Applied Biosystems, Waltham, MA, USA) DNA analyzer to identify the gene variants. FinchTV, NCBI (BlastN and BlastP), ExPASy, and ClustalW2 programs were used to analyze the gene variants.

### 4.8. Quality Control (QC) Analysis

The following QC strains were obtained from the American Type Culture Collection (ATCC): colistin-susceptible (Col-S) *E. coli* (25922) and Col-R *Proteus mirabilis* (25933); ESBL-positive *K. pneumoniae* (700603) and ESBL-negative *E. coli* (25922); and *E. coli* (BAA-2469) was used as an NDM-1.

### 4.9. Data Analysis

Data analysis was performed using the GraphPad Prism 8.0.2, IBM SPSS v.26, and BioVinci 3.0.0. A *p*-value of <0.05 was considered significant and descriptive statistics were used for the variables.

## 5. Conclusions

The study reports the emergence of *mcr-1* and *mcr-2* in several clinical isolates, including a high number of isolates that co-expressed *bla*_CTM-1_, *bla*_CTM-15_, *bla*_CMY-2_, *bla*_NDM-1_, *bla*_NDM-5_, and a few other β-lactamases. The detection of the *mcr-2* gene variant in *K. pneumoniae* is a rarely reported finding. The co-expression of diverse gene variants among β-lactamase classes was well-supported by the simultaneous occurrence of *Int-1* and *Int-2,* which can carry several drug-resistant gene cassettes. The molecular epidemiology of the co-expression of *mcr* and β-lactamases accentuates the increasing emergence of XDR clinical strains, which are difficult to treat and pose the massive threat of the clonal dissemination of these genes. Although we were able to identify therapeutic alternatives for each of the strains isolated in this study, our findings raise the question of how much time remains before a strain develops resistance against every available antimicrobial option. This situation represents a real danger to human lives and requires implacable surveillance, infection control, and the development of novel therapeutic regimens.

## Figures and Tables

**Table 1:** Characteristics of cases infected with colistin-resistant (Col-R) and *mcr*-positive gram-negative bacterial strains.

Characteristic	Col-R (*n* = 57) *n* (%)	*mcr* Detected (*n* = 19) *n* (%)	*p*-Value
Sex			
Male	19 (33.3)	7 (36.8)	0.78
Female	38 (66.7)	12 (63.2)	
Wards			
Medical ward	18 (31.6)	5 (26.3)	0.16
ICU	7 (12.3)	5 (26.3)	0.01
Surgery	6 (10.5)	4 (21.1)	0.01
Nephrology	16 (28.1)	3 (15.8)	0.74
OPD	9 (15.8)	1 (5.3)	0.71
Orthopedic	1 (1.8)	1 (5.3)	0.12
Sources			
Urine	32 (56.1)	6 (31.6)	0.55
Pus	9 (15.8)	5 (26.3)	0.01
Wound swab	10 (17.5)	4 (21.1)	0.06
Blood	4 (7)	2 (10.5)	0.38
Tracheal secretions	2 (3.5)	2 (10.5)	0.03

ICU, intensive care unit; OPD, outpatient department; Chi-square test was used to obtain *p*-values.

**Table 2:** Association between *mcr* genes and colistin-resistant (Col-R) bacterial strains (*n* = 57).

Organism	*mcr* Positive *n* (%)	*mcr* Negative *n* (%)	*p*-Value
*Escherichia coli* (*n* = 17)	9 (52.9)	8 (47.1)	0.43
*Klebsiella pneumoniae* (*n* = 9)	5 (55.6)	4 (44.4)	0.78
*Acinetobacter baumannii* (*n* = 4)	3 (75)	1 (25)	0.49
*Pseudomonas aeruginosa* (*n* = 2)	2 (100)	0 (0)	-
*Proteus mirabilis* (*n* = 25)	0 (0)	25 (100)	-

Chi-square test was used to obtain *p*-values.

**Table 3:** Demographic and clinical characteristics and the co-existence of antibiotic-resistant genes in each gram-negative *mcr*-positive bacterial.

Strain	Source	Ward	Sex	Age (years)	Organism	Detected Genes
*mcr* ST-1	Urine	Medical	Male	48	*E. coli*	*CTX-M-1, mcr-1*
*mcr* ST-2	Pus	OPD	Female	30	*K. pneumoniae*	*CTX-M-1, NDM-1, mcr-1, Int-I*
*mcr* ST-3	Pus	Nephrology	Female	60	*E. coli*	*CTX-M-15, mcr-1, Int-I*
*mcr* ST-4	Urine	ICU	Female	53	*E. coli*	*CTX-M-1, mcr-1, Int-I*
*mcr* ST-5	Pus	Surgery	Female	49	*K. pneumoniae*	*CTX-M-15, IMP, mcr-1, Int-I*
*mcr* ST-6	Urine	Medical	Male	45	*E. coli*	*CMY-2, mcr-1, Int-I*
*mcr* ST-7	Urine	Nephrology	Female	53	*E. coli*	*VIM, mcr-1, Int-I*
*mcr* ST-8	Pus	Orthopedic	Female	30	*A. baumannii*	*NDM-5, mcr-1, Int-I*
*mcr* ST-9	Wound	Surgery	Male	25	*P. aeruginosa*	*SHV-28, OXA-51, mcr-1, Int-I*
*mcr* ST-10	Tracheal secretions	ICU	Male	71	*K. pneumoniae*	*CTX-M-1, NDM-1, mcr-2, Int-2*
*mcr* ST-11	Wound	ICU	Female	65	*E. coli*	*CTX-M-11, mcr-1, Int-I*
*mcr* ST-12	Wound	ICU	Female	46	*A. baumannii*	*CTX-M-1, OXA-48, mcr-1, Int-I*
*mcr* ST-13	Blood	Nephrology	Male	43	*E. coli*	*CMY-2, mcr-1*
*mcr* ST-14	Blood	Medical	Female	20	*K. pneumoniae*	*CTX-M-10, mcr-1, Int-I*
*mcr* ST-15	Urine	Medical	Female	46	*E. coli*	*TEM-52, CTX-M-15, mcr-1, Int-I*
*mcr* ST-16	Wound	Surgery	Male	43	*K. pneumoniae*	*CTX-M-1, mcr-1, Int-I*
*mcr* ST-17	Tracheal secretions	ICU	Female	54	*A. baumannii*	*CTX-M-1, mcr-1, Int-I*
*mcr* ST-18	Urine	Medical	Male	36	*P. aeruginosa*	*CTX-M-1, CMY-2, NDM-5, mcr-1, Int-2*
*mcr* ST-19	Pus	Surgery	Female	52	*E. coli*	*SHV-12, mcr-1, Int-I*

**Table 4:** Comparison of the MIC_50_ and MIC_90_ (µg/mL) values among the bacterial strains harboring *mcr* genes.

Antibiotic	*E. coli* (*n* = 9)			*K. pneumoniae* (*n* = 5)			*A. baumannii* (*n* = 3)			*P. aeruginosa* (*n* = 2)		
	Breakpoints	MIC_50_	MIC_90_	Breakpoints	MIC_50_	MIC_90_	Breakpoints	MIC_50_	MIC_90_	Breakpoints	MIC_50_	MIC_90_
Colistin	≥4	12	24	≥4	12	32	≥4	8	12	≥4	32	64
Amikacin	≥64	2	64	≥64	64	64	≥64	128	128	≥64	2	2
Gentamicin	≥16	16	32	≥16	32	32	≥16	32	32	≥16	2	2
Co-amoxiclav	≥32/16	32/16	64/32	≥32/16	32/16	64/32	≥32/16	≥128/64	≥128/64	≥32/16	≥128/64	≥128/64
Cefoxitin	≥32	4	≥128	≥32	64	64	≥32	≥128	≥128	≥32	≥128	≥128
Aztreonam	≥16	64	≥128	≥16	≥128	≥128	≥32	≥128	≥128	≥32	≥128	≥128
Cefuroxime	≥32	≥128	≥128	≥32	≥128	≥128	≥32	≥128	≥128	≥32	≥128	≥128
Ceftriaxone	≥4	64	≥128	≥4	≥128	≥128	≥64	≥128	≥128	≥64	≥128	≥128
Cefotaxime	≥4	64	≥128	≥4	≥128	≥128	≥64	≥128	≥128	≥64	≥128	≥128
Ceftazidime	≥16	64	≥128	≥16	≥128	≥128	≥32	≥128	≥128	≥32	2	32
Cefepime	≥16	32	64	≥16	≥128	≥128	≥32	≥128	≥128	≥32	2	8
Co-trimoxazole	≥4/76	4/76	8/152	≥4/76	2/38	4/76	≥4/76	4/76	16/304	≥4/76	32/608	64/1216
Ciprofloxacin	≥1	16	≥64	≥1	32	32	≥4	≥64	≥64	≥2	0.25	4
Levofloxacin	≥2	8	32	≥2	32	32	≥8	≥64	≥64	≥4	0.5	0.5
Piperacillin-tazobactam	≥128/4	8/4	512/4	≥128/4	128/4	256/4	≥128/4	256/4	512/4	≥128/4	256/4	512/4
Imipenem	≥4	1	16	≥4	64	64	≥8	≥128	≥128	≥8	64	≥128
Meropenem	≥4	1	32	≥4	64	64	≥8	≥128	≥128	≥8	≥128	≥128
Doripenem	≥4	1	32	≥4	64	64	≥8	≥128	≥128	≥8	≥128	≥128
Tigecycline	≥2	0.5	1	≥2	8	16	≥8	1	1	≥8	≥128	≥128

## Data Availability

Data are contained within the article.

## References

[1] Liu Y.Y., Wang Y., Walsh T.R., Yi L.X., Zhang R., Spencer J., Doi Y., Tian G., Dong B., Huang X. (2016). Emergence of plasmid-mediated colistin resistance mechanism MCR-1 in animals and human beings in China: A microbiological and molecular biological study. Lancet Infect. Dis..

[2] MacNair C.R., Stokes J.M., Carfrae L.A., Fiebig-Comyn A.A., Coombes B.K., Mulvey M.R., Brown E.D. (2018). Overcoming *mcr*-1 mediated colistin resistance with colistin in combination with other antibiotics. Nat. Commun..

[3] Temkin E., Adler A., Lerner A., Carmeli Y. (2014). Carbapenem-resistant Enterobacteriaceae: Biology, epidemiology, and management. Ann. N. Y. Acad. Sci..

[4] Schwarz S., Johnson A.P. (2016). Transferable resistance to colistin: A new but old threat. J. Antimicrob. Chemother..

[5] Olaitan A.O., Morand S., Rolain J.M. (2014). Mechanisms of polymyxin resistance: Acquired and intrinsic resistance in bacteria. Front. Microbiol..

[6] Ling Z., Yin W., Shen Z., Wang Y., Shen J., Walsh T.R. (2020). Epidemiology of mobile colistin resistance genes *mcr*-1 to *mcr*-9. J. Antimicrob. Chemother..

[7] Malhotra-Kumar S., Xavier B.B., Das A.J., Lammens C., Butaye P., Goossens H. (2016). Colistin resistance gene *mcr*-1 harboured on a multidrug resistant plasmid. Lancet Infect. Dis..

[8] Javed H., Saleem S., Zafar A., Ghafoor A., Shahzad A.B., Ejaz H., Junaid K., Jahan S. (2020). Emergence of plasmid-mediated *mcr* genes from Gram-negative bacteria at the human-animal interface. Gut Pathog..

[9] Ejaz H. (2020). Dissemination of SHV, TEM and CTX-M Genotypes in Pseudomonas aeruginosa: A Pre-eminent Reason for Therapeutic Failure in Pediatrics. Ann. Clin. Lab. Sci..

[10] Manchanda V., Singh N.P. (2003). Occurrence and detection of AmpC beta-lactamases among Gram-negative clinical isolates using a modified three-dimensional test at Guru Tegh Bahadur Hospital, Delhi, India. J. Antimicrob. Chemother..

[11] van Duin D., Doi Y. (2015). Outbreak of Colistin-Resistant, Carbapenemase-Producing Klebsiella pneumoniae: Are We at the End of the Road?. J. Clin. Microbiol..

[12] Li X., Mu X., Zhang P., Zhao D., Ji J., Quan J., Zhu Y., Yu Y. (2018). Detection and characterization of a clinical Escherichia coli ST3204 strain coproducing NDM-16 and MCR-1. Infect. Drug Resist..

[13] Ejaz H., Alzahrani B., Hamad M.F.S., Abosalif K.O.A., Junaid K., Abdalla A.E., Elamir M.Y.M., Aljaber N.J., Hamam S.S.M., Younas S. (2020). Molecular Analysis of the Antibiotic Resistant NDM-1 Gene in Clinical Isolates of Enterobacteriaceae. Clin. Lab..

[14] Wang Y., Tian G.B., Zhang R., Shen Y., Tyrrell J.M., Huang X., Zhou H., Lei L., Li H.Y., Doi Y. (2017). Prevalence, risk factors, outcomes, and molecular epidemiology of *mcr*-1-positive Enterobacteriaceae in patients and healthy adults from China: An epidemiological and clinical study. Lancet Infect. Dis..

[15] Saavedra S.Y., Diaz L., Wiesner M., Correa A., Arévalo S.A., Reyes J., Hidalgo A.M., de la Cadena E., Perenguez M., Montaño L.A. (2017). Genomic and Molecular Characterization of Clinical Isolates of Enterobacteriaceae Harboring *mcr*-1 in Colombia, 2002 to 2016. Antimicrob. Agents Chemother..

[16] Bayram Y., Parlak M., Aypak C., Bayram I. (2013). Three-year review of bacteriological profile and antibiogram of burn wound isolates in Van, Turkey. Int. J. Med. Sci..

[17] Gill M.M., Usman J., Kaleem F., Hassan A., Khalid A., Anjum R., Fahim Q. (2011). Frequency and antibiogram of multi-drug resistant Pseudomonas aeruginosa. J. Coll. Physicians Surg. Pak..

[18] Hameed F., Khan M.A., Muhammad H., Sarwar T., Bilal H., Rehman T.U. (2019). Plasmid-mediated *mcr*-1 gene in Acinetobacter baumannii and Pseudomonas aeruginosa: First report from Pakistan. Rev. Soc. Bras. Med. Trop..

[19] Aghapour Z., Hasani A., Aghazadeh M., Rezaee M.A., Ganbarov K., Pourlak T., Gholizadeh P., Asgharzadeh M., Tanomand A., Kafil H. (2019). Genes involved in colistin resistance of gram-negative isolates in the northwest of Iran. Gene Rep..

[20] Wise M.G., Estabrook M.A., Sahm D.F., Stone G.G., Kazmierczak K.M. (2018). Prevalence of *mcr*-type genes among colistin-resistant Enterobacteriaceae collected in 2014-2016 as part of the INFORM global surveillance program. PLoS ONE.

[21] Elbediwi M., Li Y., Paudyal N., Pan H., Li X., Xie S., Rajkovic A., Feng Y., Fang W., Rankin S.C. (2019). Global Burden of Colistin-Resistant Bacteria: Mobilized Colistin Resistance Genes Study (1980–2018). Microorganisms.

[22] Wang R., van Dorp L., Shaw L.P., Bradley P., Wang Q., Wang X., Jin L., Zhang Q., Liu Y., Rieux A. (2018). The global distribution and spread of the mobilized colistin resistance gene *mcr*-1. Nat. Commun..

[23] Yoon E.J., Hong J.S., Yang J.W., Lee K.J., Lee H., Jeong S.H. (2018). Detection of *mcr*-1 Plasmids in Enterobacteriaceae Isolates from Human Specimens: Comparison With Those in Escherichia coli Isolates From Livestock in Korea. Ann. Lab. Med..

[24] Faccone D., Rapoport M., Albornoz E., Celaya F., De Mendieta J., De Belder D., Lucero C., Gomez S., Danze D., Pasteran F. (2020). Plasmidic resistance to colistin mediated by *mcr*-1 gene in Escherichia coli clinical isolates in Argentina: A retrospective study, 2012–2018. Rev. Panam. Salud Publica.

[25] Moosavian M., Emam N. (2019). The first report of emerging mobilized colistin-resistance (*mcr*) genes and ERIC-PCR typing in Escherichia coli and Klebsiella pneumoniae clinical isolates in southwest Iran. Infect. Drug Resist..

[26] Giske C.G. (2015). Contemporary resistance trends and mechanisms for the old antibiotics colistin, temocillin, fosfomycin, mecillinam and nitrofurantoin. Clin. Microbiol. Infect..

[27] Baraka A., Traglia G.M., Montaña S., Tolmasky M.E., Ramirez M.S. (2020). An Acinetobacter non-baumannii Population Study: Antimicrobial Resistance Genes (ARGs). Antibiotics.

[28] Lin D.L., Traglia G.M., Baker R., Sherratt D.J., Ramirez M.S., Tolmasky M.E. (2020). Functional Analysis of the Acinetobacter baumannii XerC and XerD Site-Specific Recombinases: Potential Role in Dissemination of Resistance Genes. Antibiotics.

[29] Thirapanmethee K., Srisiri A.N.T., Houngsaitong J., Montakantikul P., Khuntayaporn P., Chomnawang M.T. (2020). Prevalence of OXA-Type β-Lactamase Genes among Carbapenem-Resistant Acinetobacter baumannii Clinical Isolates in Thailand. Antibiotics.

[30] El-Mokhtar M.A., Daef E., Mohamed Hussein A.A.R., Hashem M.K., Hassan H.M. (2021). Emergence of Nosocomial Pneumonia Caused by Colistin-Resistant Escherichia coli in Patients Admitted to Chest Intensive Care Unit. Antibiotics.

[31] Reeves C.M., Magallon J., Rocha K., Tran T., Phan K., Vu P., Yi Y., Oakley-Havens C.L., Cedano J., Jimenez V. (2020). Aminoglycoside 6’-N-acetyltransferase Type Ib [AAC(6′)-Ib]-Mediated Aminoglycoside Resistance: Phenotypic Conversion to Susceptibility by Silver Ions. Antibiotics.

[32] Zheng B., Dong H., Xu H., Lv J., Zhang J., Jiang X., Du Y., Xiao Y., Li L. (2016). Coexistence of MCR-1 and NDM-1 in Clinical Escherichia coli Isolates. Clin. Infect. Dis..

[33] Mediavilla J.R., Patrawalla A., Chen L., Chavda K.D., Mathema B., Vinnard C., Dever L.L., Kreiswirth B.N. (2016). Colistin- and Carbapenem-Resistant Escherichia coli Harboring *mcr*-1 and blaNDM-5, Causing a Complicated Urinary Tract Infection in a Patient from the United States. mBio.

[34] Gillings M.R. (2014). Integrons: Past, present, and future. Microbiol. Mol. Biol. Rev..

[35] Association W.M. WMA Declaration of Helsinki–Ethical Principles for Medical Research Involving Human Subjects. https://www.wma.net/policies-post/wma-declaration-of-helsinki-ethical-principles-for-medical-research-involving-human-subjects/.

[36] CLSI (2020). Performance Standards for Antimicrobial Susceptibility Testing.

[37] Amin H., Zafar A., Ejaz H., Jameel N.U. (2013). Phenotypic characterization of ESBL producing Enterobacter cloacae among children. Pak. J. Med. Sci..

[38] Ejaz H., Younas S., Abosalif K.O.A., Junaid K., Alzahrani B., Alsrhani A., Abdalla A.E., Ullah M.I., Qamar M.U., Hamam S.S.M. (2021). Molecular analysis of blaSHV, blaTEM, and blaCTX-M in extended-spectrum β-lactamase producing Enterobacteriaceae recovered from fecal specimens of animals. PLoS ONE.

[39] Younas S., Ejaz H., Zafar A., Ejaz A., Saleem R., Javed H. (2018). AmpC beta-lactamases in Klebsiella pneumoniae: An emerging threat to the paediatric patients. J. Pak. Med. Assoc..

[40] Javed H., Ejaz H., Zafar A., Rathore A.W. (2016). Metallo-beta-lactamase producing Escherichia coli and Klebsiella pneumoniae: A rising threat for hospitalized children. J. Pak. Med. Assoc..

[41] Qamar M.U., Ejaz H., Walsh T.R., Shah A.A., Al Farraj D.A., Alkufeidy R.M., Alkubaisi N.A., Saleem S., Jahan S. (2021). Clonal relatedness and plasmid profiling of extensively drug-resistant New Delhi metallo-β-lactamase-producing Klebsiella pneumoniae clinical isolates. Future Microbiol..

[42] Rebelo A.R., Bortolaia V., Kjeldgaard J.S., Pedersen S.K., Leekitcharoenphon P., Hansen I.M., Guerra B., Malorny B., Borowiak M., Hammerl J.A. (2018). Multiplex PCR for detection of plasmid-mediated colistin resistance determinants, *mcr*-1, *mcr*-2, *mcr*-3, *mcr*-4 and *mcr*-5 for surveillance purposes. Euro Surveill.

[43] Lv D., Duan R., Fan R., Mu H., Liang J., Xiao M., He Z., Qin S., Yang J., Jing H. (2021). bla(NDM) and *mcr*-1 to *mcr*-5 Gene Distribution Characteristics in Gut Specimens from Different Regions of China. Antibiotics.

[44] Mohd Khari F.I., Karunakaran R., Rosli R., Tee Tay S. (2016). Genotypic and Phenotypic Detection of AmpC β-lactamases in Enterobacter spp. Isolated from a Teaching Hospital in Malaysia. PLoS ONE.

